# Supplementary material for: Canonical and non-canonical integrin-based adhesions dynamically interconvert
Source: Nat Commun. 2024 Mar 7;15:2093. doi: 10.1038/s41467-024-46381-x (PMC10920918; doi:10.1038/s41467-024-46381-x)
Supplement: Supplementary file 1 — Supplementary Information [file 41467_2024_46381_MOESM1_ESM.pdf]

Supplementary Material

Canonical and non-canonical integrin-based adhesions dynamically interconvert

Fabian Lukas, Claudia Matthaeus, Tania López-Hernández, Ines Lahmann, Nicole Schultz, Martin Lehmann, Dmytro Puchkov, Jan Pielage, Volker Haucke, Tanja Maritzen

Supplementary Fig.1

Supplementary Fig.2

Supplementary Fig.3

Supplementary Fig.4

Supplementary Fig.5

Supplementary Fig.6

Supplementary Fig.7

Supplementary Fig.8

Supplementary Fig.9

Supplementary Fig.10

Supplementary Fig.11

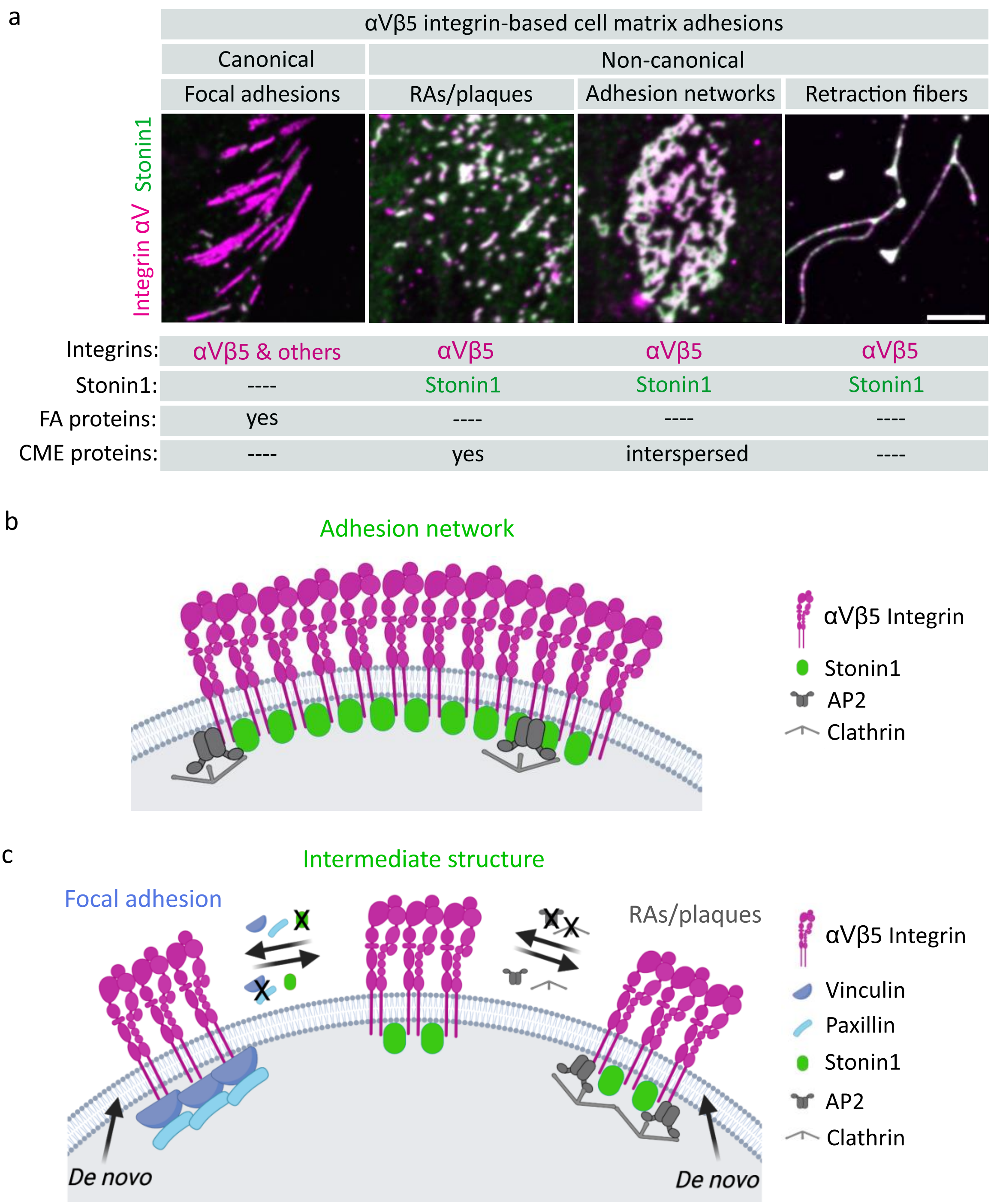

Supplementary Fig.1

Scheme of different αVβ5 integrin adhesion types and their composition and interconversion

(a) Table illustrating shapes of adhesion types and providing the composition of the different adhesion types in regards to integrins, focal adhesion proteins, endocytic proteins and stonin1. Representative merged images of C2C12 cells immunolabeled with antibodies specific for integrin αV and stonin1. Scale bars, 5 µm. FA, focal adhesions, CME, clathrin-mediated endocytosis. **(b)** Scheme of αVβ5 integrin adhesion network illustrating that stonin1 is a major component while typical endocytic proteins are only present in an interspersed manner. **(c)** Scheme illustrating the dynamic relationships between the different adhesion types. While focal adhesions and RAs/plaques can form de novo, they can also be generated by conversion of an existing αVβ5 integrin- and stonin1-positive adhesion. Recruitment of endocytic proteins gives rise to RAs/plaques while loss of stonin1 and concomitant recruitment of focal adhesion proteins such as paxillin and vinculin results in focal adhesions. Both conversions can also proceed in the reverse manner.

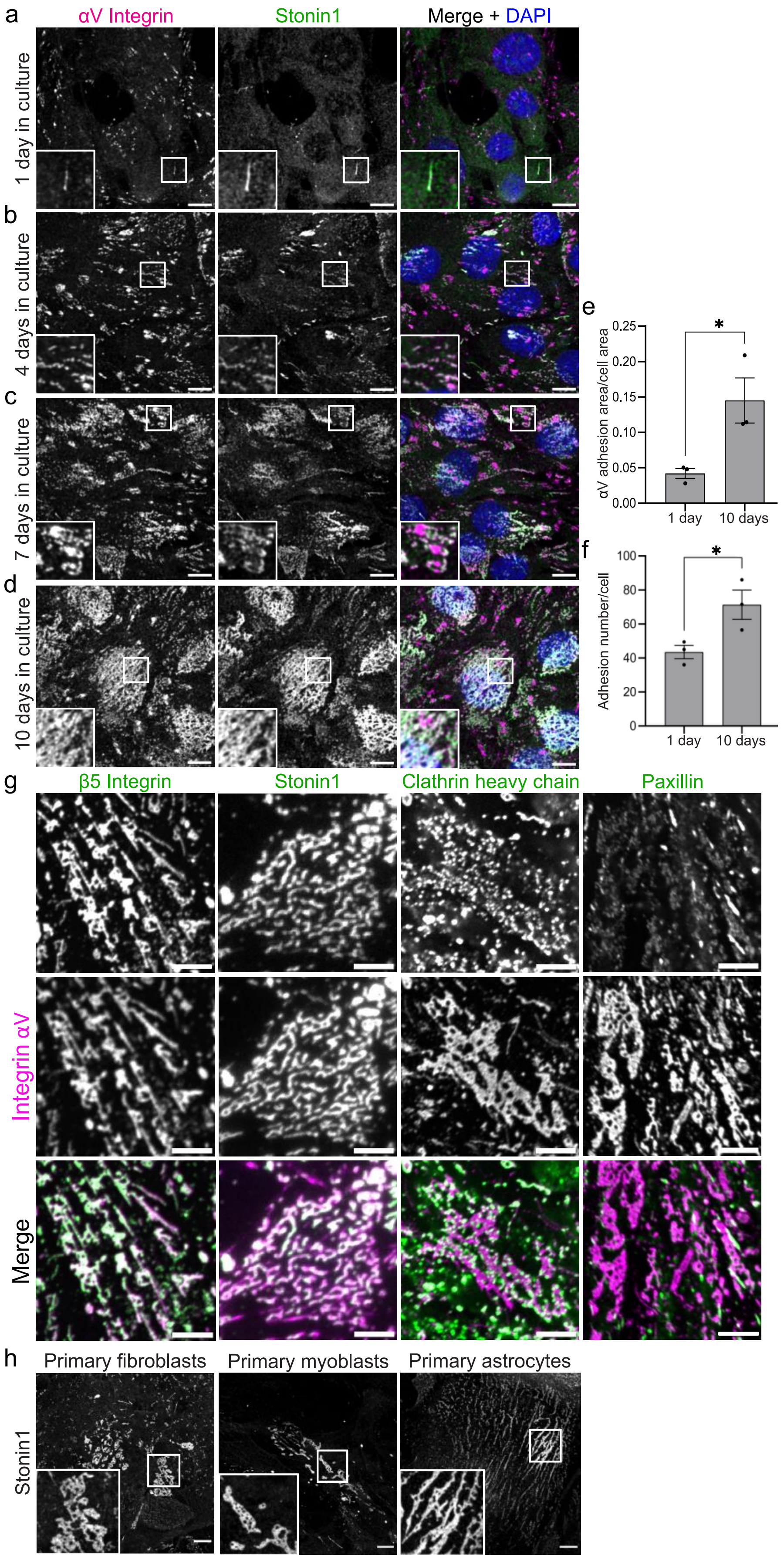

Supplementary Fig.2

αVβ5-positive non-canonical adhesions can grow into extensive stonin1-positive networks

(a-d) Extended long-term culture promotes generation of extensive integrin αV- and stonin1-positive networks. C2C12 cells were cultured for 1 (a), 4 (b), 7 (c) or 10 (d) days on vitronectin,

fixed and immunolabeled for αV integrin and stonin1. Nuclei were stained with DAPI. Scale bar, 10 µm. **(e-f)** Quantification of mean αV integrin adhesion size per cell (e) and percentage of αV integrin adhesion-covered cell area (f) between 1 and 10 days in culture (mean±SEM, N=3 independent experiments, two-tailed unpaired Student´s t-test, (e)*=p=0.0347, (f) *=p=0.0413). **(g)** Extensive integrin αVβ5- and stonin1-positive adhesion networks are also present in retinal pigment epithelial cells. hTERT-RPE1 cells were grown for 72 h on collagen, fixed and immunolabeled with antibodies against αV integrin in combination with antibodies against either β5 integrin, stonin1, clathrin heavy chain or paxillin. Scale bar, 10 µm. **(h)** Extensive stonin1-positive adhesion networks can also be found in a range of primary cells. Primary fibroblasts, myoblasts and astrocytes grown in long-term culture were fixed and immunolabeled for stonin1. Scale bar, 10 µm. Source data are provided as a Source Data file.

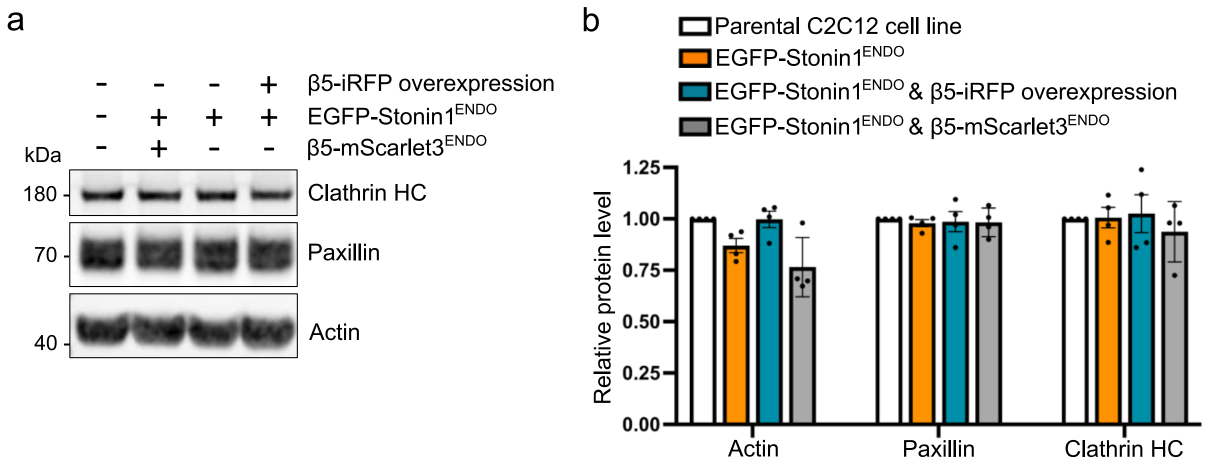

Supplementary Fig.3

Unaltered expression levels of canonical and non-canonical adhesion components in genome-edited cells

(a,b) Lysates of the parental C2C12 line, of the cell line expressing endogenously tagged EGFP-stonin1, of the doubly genome-edited cell line expressing endogenously tagged EGFP-stonin1 and integrin β5-mScarlet3 and of the cell line expressing endogenously tagged EGFP-stonin1 and transduced with β5-iRFP were subjected to immunoblotting with the indicated antibodies. **(a)** Representative immunoblot. **(b)** Quantification of relative protein levels. Results of edited cell lines were normalized to results of the parental cell line. None of the tested proteins exhibited significant changes compared to the parental C2C12 cell line (mean±SEM, N=4 independent experiments, Kruskall-Wallis test with Dunn´s multiple comparison test). Source data are provided as a Source Data file.

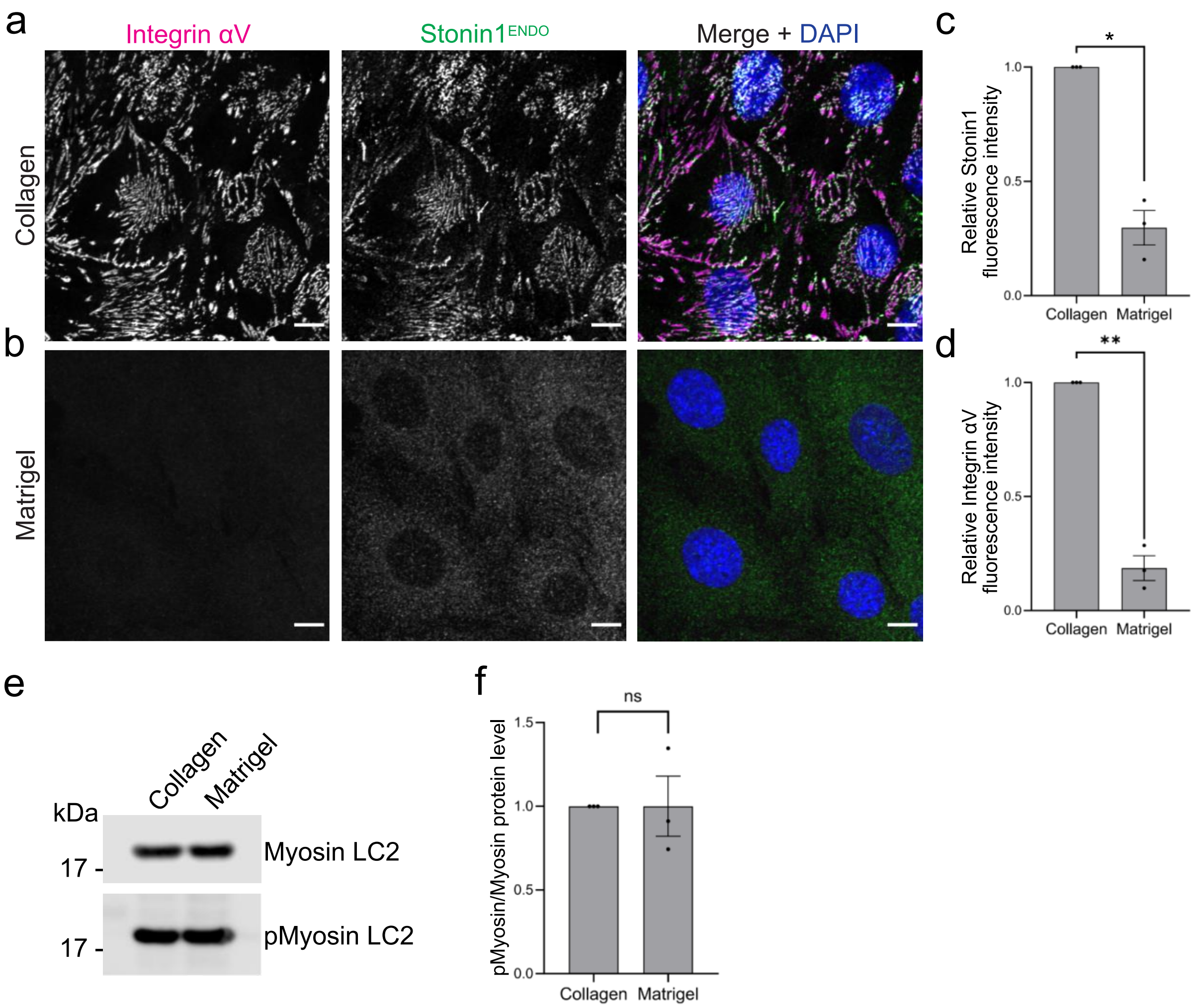

Supplementary Fig.4

Collagen promotes the generation of large αV integrin-positive adhesion networks

(a-d) C2C12 cells were grown for 24 h on collagen I- (a) or Matrigel-coated (b) cover slips, fixed and immunolabeled for αV integrin and EGFP. Nuclei were stained with DAPI. Scale bar, 10 µm. Quantification of stonin1 (c) and αV integrin (d) fluorescence intensity under the nucleus for cells seeded on collagen or Matrigel reveals significantly lower fluorescence signals for both upon Matrigel coating (mean±SEM, N=3 independent experiments, One-sample t-test, *=p=0.0113, **=p=0.0044). **(e-f)** Cellular tension does not significantly differ between cells grown on collagen I or Matrigel based on phospho-myosin light chain 2 levels. C2C12 cells were grown overnight on collagen I- or Matrigel-coated dishes. Cell lysates were subjected to immunoblotting with myosin and phospho-myosin light chain 2-specific antibodies. (e) Representative immunoblot. (f) Quantification of phospho-myosin light chain 2 over myosin light chain 2 protein levels (mean±SEM, N=3 independent experiments, One-sample t-test, ns = non significant). Source data are provided as a Source Data file.

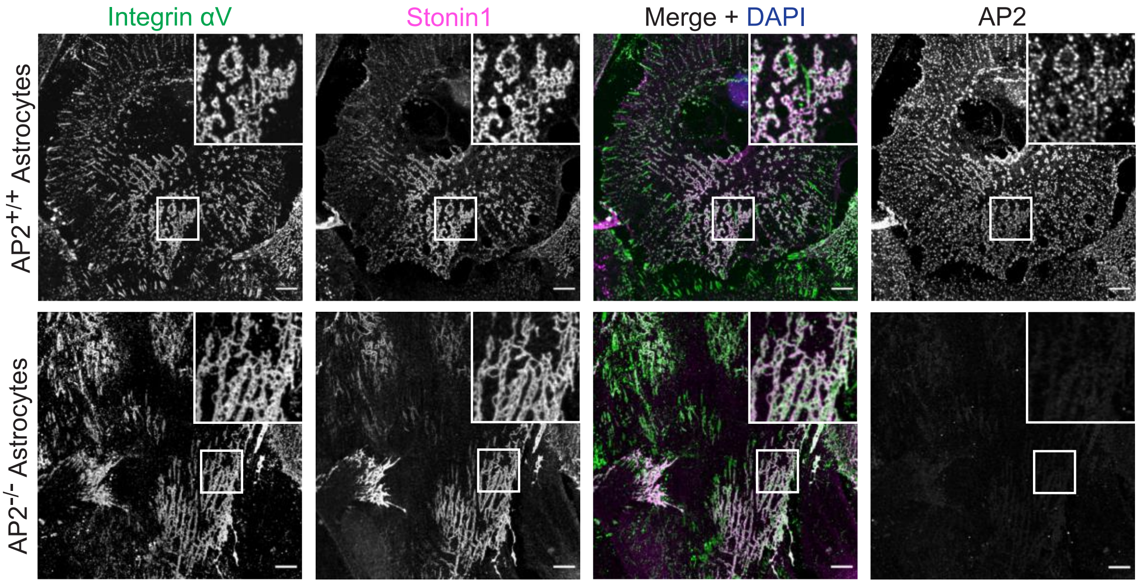

Supplementary Fig.5

αV/stonin1-positive networks persist in absence of AP2

Confocal images of primary AP2 wildtype and knockout astrocytes fixed and immunolabeled for stonin1, αV integrin and AP2. Scale bars, 10 µm.

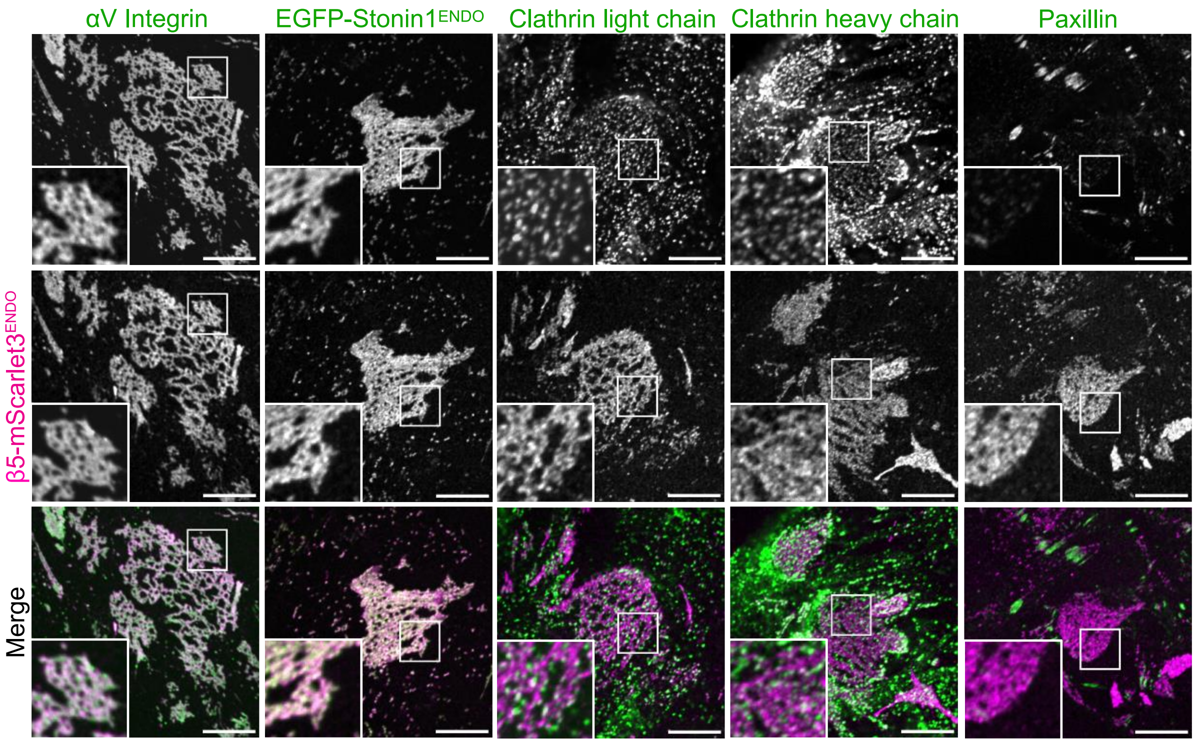

Supplementary Fig.6

αVβ5/stonin1-positive networks are also present in C2C12 cells expressing genome-edited integrin β5-mScarlet3

C2C12 cells expressing endogenously tagged integrin β5-mScarlet3 and endogenously tagged EGFP-stonin1 were grown on collagen for 72 h, fixed and immunolabeled with antibodies against the proteins indicated on top of the images. Scale bars, 10 µm. Consistent with our results in cells overexpressing integrin β5-iRFP, αVβ5 integrins form extensive adhesion networks which show a large overlap with stonin1, but only a limited, mostly punctate co-localization with the endocytic markers clathrin light chain and clathrin heavy chain.

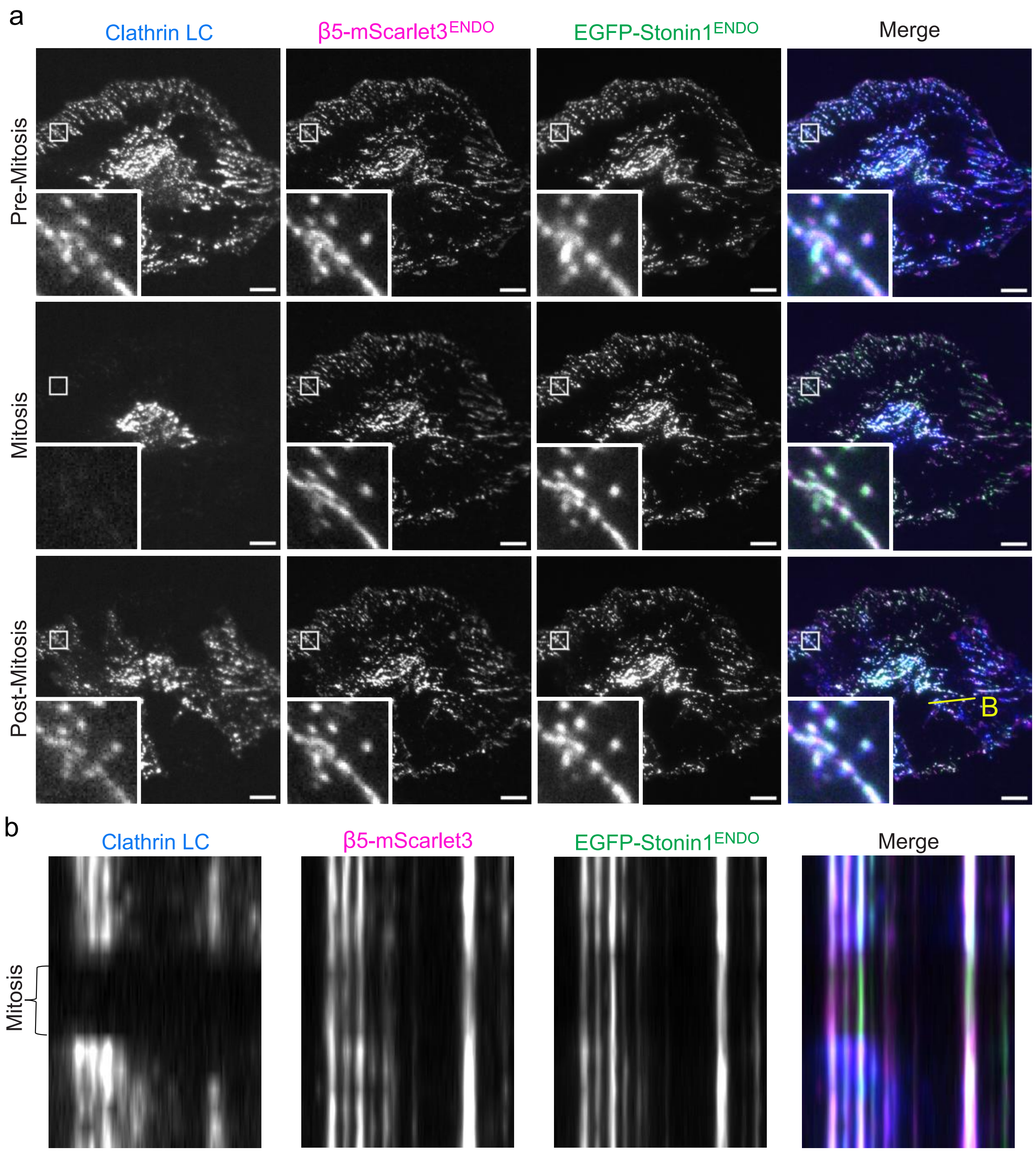

Supplementary Fig.7

Remodeling of RAs/plaques during mitosis in cells expressing endogenously tagged integrin β5-mScarlet3 entails loss of clathrin

(a) C2C12 cells expressing endogenously tagged integrin β5-mScarlet3 and endogenously tagged EGFP-stonin1 and stably transduced with mRFP-clathrin light chain (LC) were cultured for 24 h and then subjected to TIRF live cell microscopy. Scale bars, 10 µm. **(b)** 150 min kymograph along the line indicated in (a) reveals loss of clathrin from β5 integrin scaffolds anchoring mitotic retraction fibers during mitotic cell rounding.

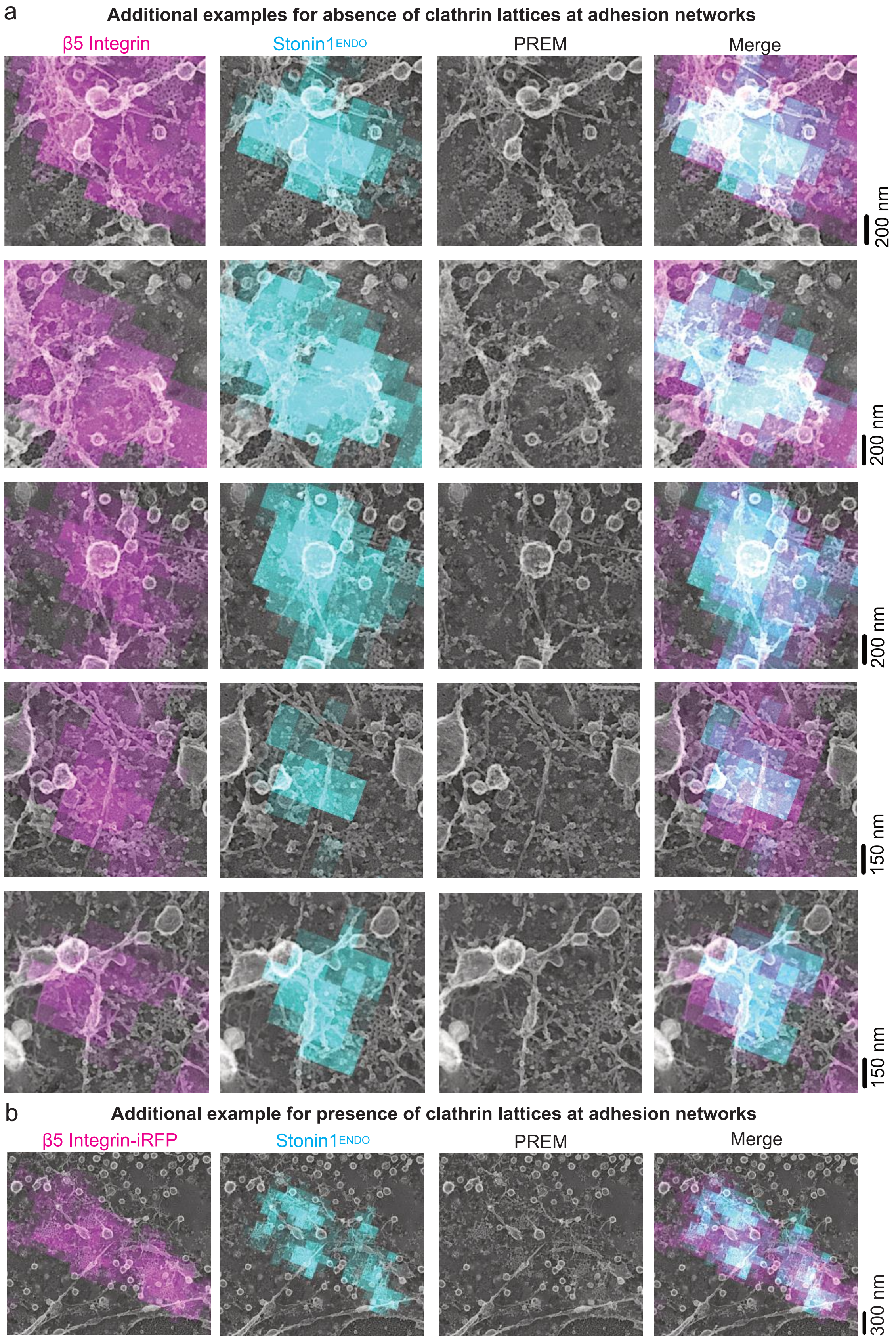

Supplementary Fig.8

Supplementary data to Fig.4

(a,b) C2C12 cells expressing genome-edited EGFP-stonin1 and stably transduced with β5 integrin-iRFP were grown for 48 h on collagen and subjected to correlated light and electron microscopy after unroofing. **(a)** Additional examples for absence of clathrin lattices at β5 integrin- and stonin1-positive adhesion networks. **(b)** Additional example for presence of clathrin lattices at β5 integrin- and stonin1-positive adhesion networks.

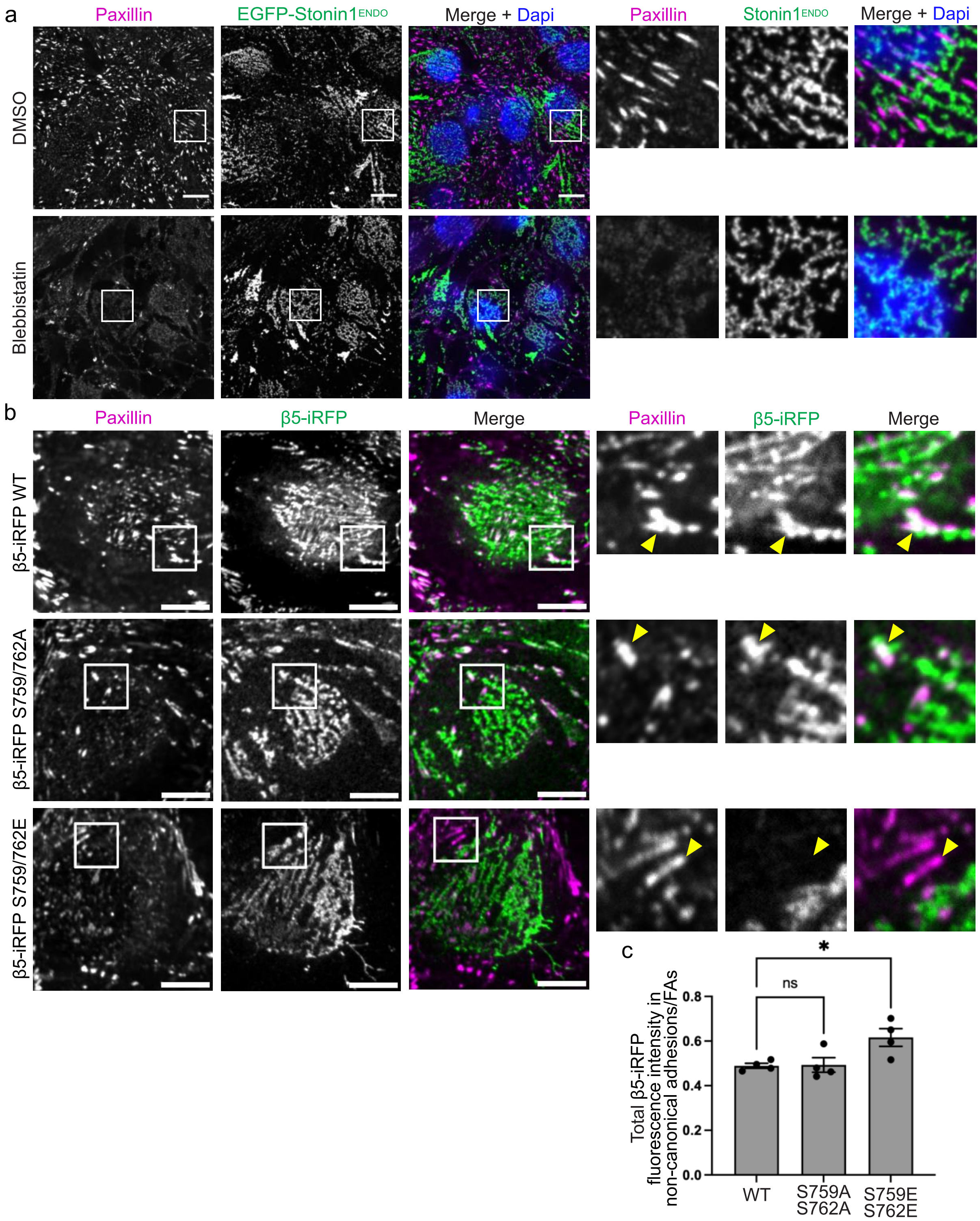

Supplementary Fig.9

Determinants for the formation and maintenance of large non-canonical adhesion networks

(a) αVβ5 integrin adhesion networks persist upon loss of actin-dependent tension in contrast to focal adhesions. C2C12 cells expressing genome-edited EGFP-stonin1 were seeded on collagen and treated with the myosin II inhibitor blebbistatin or the vehicle DMSO for 1 h. Fixed cells were immunolabeled for EGFP and paxillin and analyzed by TIRF microscopy. Nuclei were stained with DAPI. Scale bars, 10 µm. **(b)** Integrin β5 displays less localization at focal adhesions upon a phospho-mimetic mutation of its SERS motif (S759E/S762E), while WT integrin β5 and a phospho-dead SERS motif mutant (S759A/S762A) are more frequently also found in focal adhesions. C2C12 cells were stably transduced with either β5-iRFP WT, β5-iRFP S759E/S762E or β5-iRFP S759A/S762A. Cells were fixed and stained for paxillin to label

focal adhesions. Scale bars, 10 µm. **(c)** Quantification of the ratio of total β5-iRFP fluorescence intensity in non-canonical adhesions/focal adhesions. Non-canonical adhesions were segmented via their stonin1 staining, and focal adhesions were segmented via their paxillin staining (mean±SEM, N=4 independent experiments, One-way ANOVA followed by Dunnett´s multiple comparison test, *=p=0.0289). Source data are provided as a Source Data file.

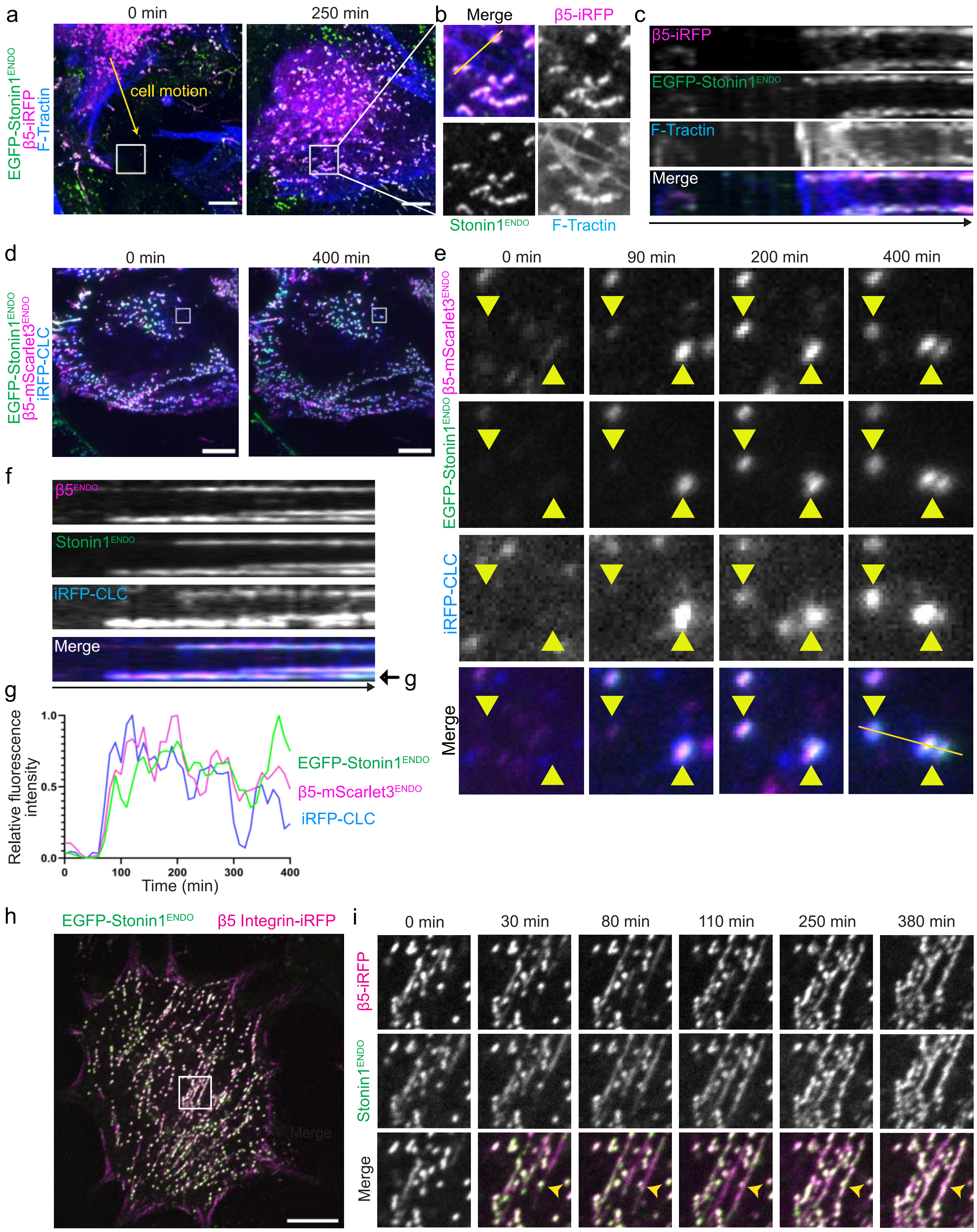

Supplementary Fig.10

RAs/plaques can arise de novo and can fuse into larger networks

(a-g) RAs/plaques can arise de novo. (a-c) C2C12 cells expressing genome-edited EGFP-stonin1 and stably transduced with β5 integrin-iRFP and mCherry-F-Tractin were cultured for 48 h and subjected to 250 min live-cell confocal spinning disk microscopy. (a) Overview of a representative migrating cell at the start and after 250 min. Scale bars, 10 µm. (b) Magnification of inset at 250 min showing newly formed RAs/plaques. (c) The kymographs along the line indicated in the magnification in (b) reveal that β5 integrin and stonin1 simultaneously assemble into new RAs/plaques which are also enriched in actin. (d-g) Clathrin is co-recruited when RAs/plaques arise de novo. C2C12 cells expressing genome-edited β5-integrin-mScarlet3 and genome-edited EGFP-stonin1 and stably transduced with iRFP-clathrin light chain were cultured for 24 h and subjected to 400 min live-cell TIRF microscopy. (d) Overview of a

representative cell at the start and after 400 min. Scale bars, 10 µm. (e) Magnification of inset for four different time points showing newly formed RAs/plaques (arrows point out examples). (f) The kymographs along the line indicated in (e) reveal that β5 integrin, stonin1 and clathrin simultaneously assemble into new RAs/plaques. (g) Line profile illustrates co-recruitment of β5 integrin, stonin1 and clathrin. **(h-i)** Initial small β5 integrin/stonin1-positive adhesions grow together into larger networks. C2C12 cells expressing genome-edited EGFP-stonin1 and stably transduced with β5 integrin-iRFP were cultured for 48 h and subjected to 430 min live-cell TIRF microscopy. (h) Overview picture at 430 min. Scale bar, 48 µm. (i) Time series of inset in (h) illustrating the slow extension of initially small adhesions into larger networks. Source data are provided as a Source Data file.

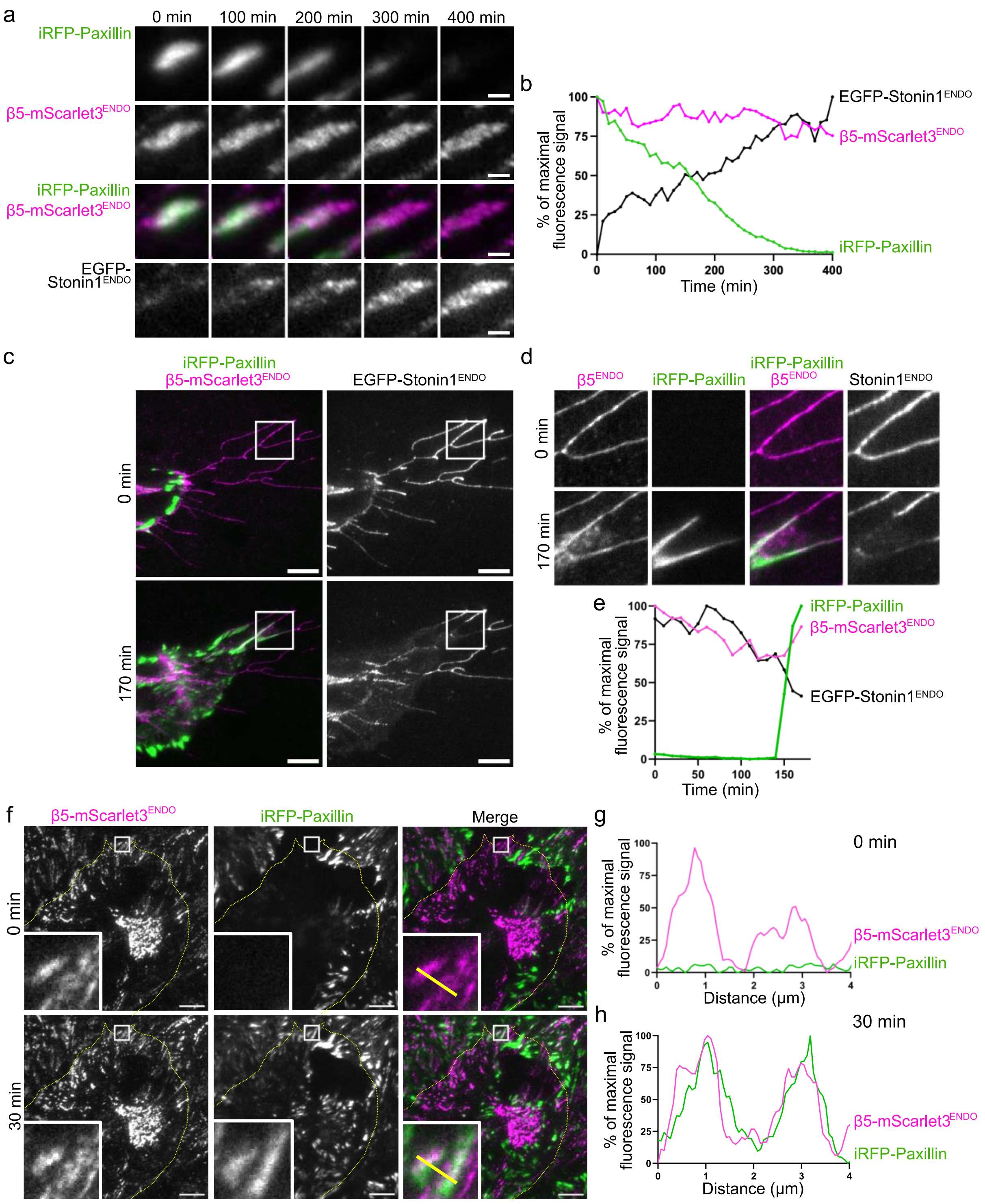

Supplementary Fig.11

Confirmation of conversion processes between canonical and non-canonical adhesions in cells expressing genome-edited integrin β5-mScarlet3

(a,b) RAs/plaques can arise during focal adhesion disassembly by recycling the existing integrin β5 scaffold. C2C12 cells expressing genome-edited integrin β5-mScarlet3 and genome-edited EGFP-stonin1 and stably transduced with iRFP-paxillin were cultured for 48 h and subjected to 400 min live cell confocal spinning disk microscopy. **(a)** Representative images showing a disassembling focal adhesion converting into an RA/plaque by losing paxillin and acquiring stonin1. Scale bar, 2 µm. **(b)** Quantification of relative fluorescence profiles of indicated proteins within β5 integrin-positive adhesion in (a) over the course of 400 min. The highest fluorescence value for each imaged protein was set to 100%, and all other fluorescence values of the same protein were expressed relative to it. **(c-e)** Focal adhesions assemble at β5 integrin-positive migratory retraction fibers. C2C12 cells expressing genome-edited integrin

β5-mScarlet3 and genome-edited EGFP-stonin1 and stably transduced with iRFP-paxillin were grown for 24 h and subjected to confocal live cell imaging. **(c)** Representative images at 0 min and 170 min. Scale bars, 10 µm. **(d)** Magnification of insets shows paxillin-negative β5 integrin-positive retraction fibers left behind by migrating cell. Magnification of inset at 170 min demonstrates recruitment of focal adhesion marker paxillin to β5 integrin scaffold and concomitant loss of stonin1 upon cellular respreading across retraction fiber. **(e)** Quantification of integrin β5-mScarlet3, EGFP-stonin1 and iRFP-paxillin fluorescence over time within the bifurcated adhesion depicted in (d). The area within which the fluorescence was quantified was based on a segmentation of the β5 staining at 0 min. The highest fluorescence value for each imaged protein over the time course was set to 100%, and all other fluorescence values of the same protein were expressed relative to it. **(f-g)** Focal adhesions assemble at β5 integrin-positive mitotic RAs/plaques. C2C12 cells expressing genome-edited integrin β5-mScarlet3 and genome-edited EGFP-stonin1 and stably transduced with iRFP-paxillin were grown for 24 h and subjected to confocal live cell imaging. **(f)** Images of a representative cell (outlined in white) during cytokinesis (0 min) and after respreading (65 min). Scale bar, 10 µm. Magnified insets at 0 min show β5 integrin-positive mitotic RAs/plaques of a cell in cytokinesis which has disassembled its focal adhesions. Magnified insets at 65 min show the recruitment of the focal adhesion marker paxillin to former mitotic RAs/plaques converting them into focal adhesions. **(g-h)** iRFP-paxillin and integrin β5-mScarlet3 fluorescence (along the line depicted in (f)) at cytokinesis (0 min) and after respreading (65 min). The highest fluorescence value for each imaged protein along the line and across both time points was set to 100%, and all other fluorescence values of the same protein were expressed relative to it. Source data are provided as a Source Data file.
