# Supplementary material for: Canonical and non-canonical integrin-based adhesions dynamically interconvert
Source: Nat Commun. 2024 Mar 7;15:2093. doi: 10.1038/s41467-024-46381-x (PMC10920918; doi:10.1038/s41467-024-46381-x)
Supplement: Supplementary file 3 — Description of Additional Supplementary Files [file 41467_2024_46381_MOESM3_ESM.pdf]

Supplementary Data 1. List of plasmids used in this study.

Supplementary Data 2. List of antibodies used in this study.

Supplementary Movie 1. Representative time-lapse imaging (to Fig. 3e) showing that the remodeling of external RAs/plaques during mitosis entails the loss of clathrin. Time interval 400 min, 1 frame per 10 min. Scale bar, 10 μm.

Supplementary Movie 2. Representative time-lapse imaging (to Fig. 5c) of a focal adhesion being remodeled into a non-canonical $\alpha V\beta 5$ integrin adhesion. Time interval 120 min, 1 frame per 10 min. Scale bar, 10 μm.

Supplementary Movie 3. Representative time-lapse imaging (to Fig. 6b) of a focal adhesion assembling at a $\beta 5$ integrin-positive migratory retraction fiber. Time interval 30 min, 1 frame per 5 min. Scale bar, 10 μm.

Supplementary Movie 4. Representative time-lapse imaging (to Fig. 6d) of a focal adhesion assembling at a $\beta 5$ integrin-positive mitotic RA/plaque. Time interval 65 min, 1 frame per 5 min. Scale bar, 10 μm.
