# Supplementary material for: Canonical and non-canonical integrin-based adhesions dynamically interconvert
Source: Nat Commun. 2024 Mar 7;15:2093. doi: 10.1038/s41467-024-46381-x (PMC10920918; doi:10.1038/s41467-024-46381-x)
Supplement: Supplementary file 10 — Reporting Summary [file 41467_2024_46381_MOESM10_ESM.pdf]

nature portfolio

Corresponding author(s): Tanja Maritzen

Last updated by author(s): Feb 9, 2024

Reporting Summary

Nature Portfolio wishes to improve the reproducibility of the work that we publish. This form provides structure for consistency and transparency in reporting. For further information on Nature Portfolio policies, see our Editorial Policies and the Editorial Policy Checklist.

Statistics

For all statistical analyses, confirm that the following items are present in the figure legend, table legend, main text, or Methods section.

n/a	Confirmed	
☐	☒	The exact sample size (*n*) for each experimental group/condition, given as a discrete number and unit of measurement
☐	☒	A statement on whether measurements were taken from distinct samples or whether the same sample was measured repeatedly
☐	☒	The statistical test(s) used AND whether they are one- or two-sided<br>*Only common tests should be described solely by name; describe more complex techniques in the Methods section.*
☒	☐	A description of all covariates tested
☐	☒	A description of any assumptions or corrections, such as tests of normality and adjustment for multiple comparisons
☐	☒	A full description of the statistical parameters including central tendency (e.g. means) or other basic estimates (e.g. regression coefficient) AND variation (e.g. standard deviation) or associated estimates of uncertainty (e.g. confidence intervals)
☐	☒	For null hypothesis testing, the test statistic (e.g. *F*, *t*, *r*) with confidence intervals, effect sizes, degrees of freedom and *P* value noted<br>*Give P values as exact values whenever suitable.*
☒	☐	For Bayesian analysis, information on the choice of priors and Markov chain Monte Carlo settings
☒	☐	For hierarchical and complex designs, identification of the appropriate level for tests and full reporting of outcomes
☐	☒	Estimates of effect sizes (e.g. Cohen's *d*, Pearson's *r*), indicating how they were calculated

Our web collection on statistics for biologists contains articles on many of the points above.

Software and code

Policy information about availability of computer code

Data collection	Quantitative Western Blot measurements were obtained with LI-COR Odyssey software, version 5.2. Confocal and TIRF images were acquired using Nikons NIS-Elements software, last version 5.30.06.
Data analysis	Microsoft Excel 365 was used for data storage and processing. GraphPad Prism (version 9) was used for statistical analysis and generation of graphs. Fiji/ImageJ (version 1.55f) and CellProfiler-3.1.9 were used for image analysis.

For manuscripts utilizing custom algorithms or software that are central to the research but not yet described in published literature, software must be made available to editors and reviewers. We strongly encourage code deposition in a community repository (e.g. GitHub). See the Nature Portfolio guidelines for submitting code & software for further information.

Data

Policy information about availability of data

All manuscripts must include a data availability statement. This statement should provide the following information, where applicable:

- Accession codes, unique identifiers, or web links for publicly available datasets
- A description of any restrictions on data availability
- For clinical datasets or third party data, please ensure that the statement adheres to our policy

All data underlying the findings presented in this paper are either contained within the paper or its supplementary files. In addition, source data are provided with this paper.

Research involving human participants, their data, or biological material

Policy information about studies with human participants or human data. See also policy information about sex, gender (identity/presentation), and sexual orientation and race, ethnicity and racism.

Reporting on sex and gender	Sex and gender were not considered in our study design since our study is based on mammalian cell lines and investigates basic cellular processes that are regarded as independent of sex and gender.
Reporting on race, ethnicity, or other socially relevant groupings	We do not use socially constructed or socially relevant categorization variables in our study.
Population characteristics	Our study does not involve human research participants.
Recruitment	Our study did not involve the recruitment of participants.
Ethics oversight	The study protocol did not need to be approved by an ethics committee being based on standard cell lines.

Note that full information on the approval of the study protocol must also be provided in the manuscript.

Field-specific reporting

Please select the one below that is the best fit for your research. If you are not sure, read the appropriate sections before making your selection.

- [x] Life sciences
- [ ] Behavioural & social sciences
- [ ] Ecological, evolutionary & environmental sciences

For a reference copy of the document with all sections, see nature.com/documents/nr-reporting-summary-flat.pdf

Life sciences study design

All studies must disclose on these points even when the disclosure is negative.

Sample size	Sample sizes were not predetermined based on pre-specified effect size, but were selected based on commonly adopted standards in the field taking into account previous experience and literature to ensure statistically meaningful comparisons and adequate statistical power. The numbers of samples analyzed are provided in the figure legends for all experiments.
Data exclusions	No samples were excluded from analysis.
Replication	For all quantified experiments, the number of independent experimental replications is provided in the figure legends. Results depicted in the form of representative images, including images of adhesion types and microscopical pictures of life-cell imaging experiments, were observed in at least three independent experiments.
Randomization	For all treatments, cultured cells of the required type were randomly allocated into control and treatment group without using a formalized randomization procedure.
Blinding	Investigators were generally not blinded as the experimental work flow required investigators to know the identity of the samples. However, data analysis occured in an unbiased manner by applying analysis routines from image analysis programs in an identical manner to all images of the different experimental groups.

Reporting for specific materials, systems and methods

We require information from authors about some types of materials, experimental systems and methods used in many studies. Here, indicate whether each material, system or method listed is relevant to your study. If you are not sure if a list item applies to your research, read the appropriate section before selecting a response.

Materials & experimental systems

n/a	Involved in the study	
☐	☒	Antibodies
☐	☒	Eukaryotic cell lines
☒	☐	Palaeontology and archaeology
☒	☐	Animals and other organisms
☒	☐	Clinical data
☒	☐	Dual use research of concern
☒	☐	Plants

Methods

n/a	Involved in the study	
☒	☐	ChIP-seq
☒	☐	Flow cytometry
☒	☐	MRI-based neuroimaging

Antibodies

Antibodies used

Primary Antibodies:
Mouse polyclonal Anti-Clathrin heavy chain; Abcam Cat# ab21679; RRID:AB_2083165 (used at 1:100 for IF and 1:1000 for WB)
Mouse monoclonal Anti-Clathrin light chain; Santa Cruz Cat# sc-12735; RRID:AB_627264 (used at 1:100 for IF)
Rabbit polyclonal Anti-Eps15R; gift from Sara Sigismund (used at 1:100 for IF)
Chicken polyconal Anti-GFP; Abcam Cat# ab13970; RRID:AB_300798 (used at 1:2000 for IF)
Rabbit polyclonal Anti-Intersectin1-SH3A-C; gift from Oleg Shupliakov (used at 1:100 for IF)
Rabbit monoclonal Anti-Paxillin; Abcam Cat# ab32084; RRID:AB_779033 (used at 1:250 for IF and 1:5000 for WB)
Rabbit polyclonal Anti-Phospho-Myosin Light Chain 2; Cell Signaling Cat# 3671; RRID:AB_330248 (used at 1:1000 for WB)
Rabbit monoclonal Anti-Myosin Light Chain 2; Cell Signaling Cat# 8505; RRID: AB_2728760 (used at 1:1000 for WB)
Rabbit polyclonal Anti-STON1-GTF2A1L; Sigma Aldrich Cat# HPA005715; RRID:AB_1080113 (used at 1:100 for IF)
Rabbit polyclonal Anti-STON2; Sigma Aldrich Cat# HPA003086; RRID:AB_2302748 (used at 1:200 for IF)
Rabbit polyclonal Anti-mouse Stonin1; selfmade (used at 1:100 for IF)
Mouse monoclonal Anti-Vinculin; Sigma Aldrich Cat# V9264; RRID:AB_10603627 (used at 1:100 for IF)
Mouse monoclonal Anti-αAdaptin; Abcam Cat# ab2730; RRID:AB_303255 (used at 1:100 for IF)
Rat monoclonal Anti-mouse CD51 (αV Integrin); BD Biosciences Cat# 550024; RRID: AB_393537 (used at 1:50 for IF)
Mouse monoclonal Anti-αV Integrin; Santa Cruz Cat# sc-376156; RRID:AB_10989554 (used at 1:100 for IF)
Mouse monoclonal Anti-β-actin; Sigma-Aldrich Cat#A5441; RRID:AB_476744 (used 1:5000 for WB)
Rabbit recombinant monoclonal Anti-β5 Integrin; Cell Signaling Cat# 3629; RRID:AB_2249358 (used at 1:100 for IF)

Secondary Antibodies:
Goat anti-chicken IgY Alexa Fluor 488, Abcam, Cat# ab150169; RRID:AB_2636803 (used at 1:400 for IF)
Goat anti-mouse IgG Alexa Fluor 488, Thermo Fisher Scientific , Cat# A11029; RRID: AB_2534088 (used at 1:400 for IF)
Goat anti-mouse IgG Alexa Fluor 568, Thermo Fisher Scientific Cat# A-11031; RRID: AB_144696 (used at 1:400 for IF)
Goat anti-mouse IgG Alexa Fluor 647, Thermo Fisher Scientific , Cat# A21236; RRID:AB_2535805 (used at 1:400 for IF)
Goat anti-mouse IgG (H + L) IRDye® 680RD, LI-COR Biosciences, Cat# 926-68070; RRID:AB_2651128 (used at 1:10000 for WB)
Goat anti-rabbit IgG Alexa Fluor 568, Thermo Fisher Scientific, Cat# A11036; RRID: AB_143157 (used at 1:400 for IF)
Donkey anti-rabbit IgG Alexa Fluor 647, Invitrogen, Cat# A31573; RRID: AB_2536183 (used at 1:400 for IF)
Goat anti-rabbit IgG IRDye® 800CW, LI-COR Biosciences, Cat# 926-32211; RRID:AB_621843 (used at 1:10000 for WB)
Donkey anti-rat IgG Alexa Fluor 488, Thermo Fisher Scientific, Cat# A-21208; RRID:AB_2535794 (used at 1:200 for IF)
Donkey anti-rat IgG Alexa Fluor 647, Dianova, Cat# 712-605-153; RRID:AB_2340694 (used at 1:200 for IF)

Validation

Anti-Clathrin heavy chain: Manufacturer-verified for use in: WB, IP, IF. Relevant citation: Soykan T, Kaempf N, Sakaba T, Vollweiter D, Goerdeler F, Puchkov D, Kononenko NL, Haucke V. Synaptic Vesicle Endocytosis Occurs on Multiple Timescales and Is Mediated by Formin-Dependent Actin Assembly. Neuron. 2017 Feb 22;93(4):854-866.e4. doi: 10.1016/j.neuron.2017.02.011. PMID: 28231467.

Anti-Clathrin light chain: Manufacturer-verified for use in: WB, IP, IF. Relevant citation: Bucher D, Frey F, Sochacki KA, Kummer S, Bergeest JP, Godinez WJ, Kräusslich HG, Rohr K, Taraska JW, Schwarz US, Boulant S. Clathrin-adaptor ratio and membrane tension regulate the flat-to-curved transition of the clathrin coat during endocytosis. Nat Commun. 2018 Mar 16;9(1):1109. doi: 10.1038/s41467-018-03533-0. PMID: 29549258; PMCID: PMC5856840.

Anti-Eps15R: Gift from Sara Sigismund. Relevant citation: Pascolutti R, Algisi V, Conte A, Raimondi A, Pasham M, Upadhyayula S, Gaudin R, Maritzen T, Barbieri E, Caldieri G, Tordonato C, Confalonieri S, Freddi S, Malabarba MG, Maspero E, Polo S, Tacchetti C, Haucke V, Kirchhausen T, Di Fiore PP, Sigismund S. Molecularly Distinct Clathrin-Coated Pits Differentially Impact EGFR Fate and Signaling. Cell Rep. 2019 Jun 4;27(10):3049-3061.e6. doi: 10.1016/j.celrep.2019.05.017. PMID: 31167147; PMCID: PMC6581797.

Anti-GFP: Manufacturer-verified for use in: WB, ICC/IF. Relevant citation: Wallroth A, Koch PA, Marat AL, Krause E, Haucke V. Protein kinase N controls a lysosomal lipid switch to facilitate nutrient signalling via mTORC1. Nat Cell Biol. 2019 Sep;21(9):1093-1101. doi: 10.1038/s41556-019-0377-3. Epub 2019 Aug 26. PMID: 31451768.

Anti-Intersectin1-SH3A-C: Gift from Oleg Shupliako. Relevant citation: Pechstein A, Bacetic J, Vahedi-Faridi A, Gromova K, Sundborger A, Tomlin N, Krainer G, Vorontsova O, Schäfer JG, Owe SG, Cousin MA, Saenger W, Shupliakov O, Haucke V. Regulation of synaptic vesicle recycling by complex formation between intersectin 1 and the clathrin adaptor complex AP2. Proc Natl Acad Sci U S A. 2010 Mar 2;107(9):4206-11. doi: 10.1073/pnas.0911073107. Epub 2010 Feb 16. PMID: 20160082; PMCID: PMC2840162.

Anti-Paxillin: Manufacturer-verified for use in: IHC-P, IP, WB, ICC/IF. Relevant citation: Lawson C, Lim ST, Uryu S, Chen XL, Calderwood DA, Schlaepfer DD. FAK promotes recruitment of talin to nascent adhesions to control cell motility. J Cell Biol. 2012 Jan 23;196(2):223-32. doi: 10.1083/jcb.201108078. Erratum in: J Cell Biol. 2012 Feb 6;196(3):387. PMID: 22270917; PMCID: PMC3265949.

Anti-Phospho-Myosin Light Chain 2 Cell Signaling Cat# 3671: Manufacturer-verified for use in: WB. Relevant citation: Zuidema A, Wang W, Kreft M, Bleijerveld OB, Hoekman L, Aretz J, Böttcher RT, Fässler R, Sonnenberg A. Molecular determinants of αVβ5 localization in flat clathrin lattices - role of αVβ5 in cell adhesion and proliferation. J Cell Sci. 2022 Jun 1;135(11):jcs259465. doi: 10.1242/jcs.259465. Epub 2022 Jun 6. PMID: 35532004; PMCID: PMC9234671.

Anti-Myosin Light Chain 2: Manufacturer-verified for use in: WB. Relevant citation: Fan T, Hou Y, Ge W, Fan T, Feng X, Guo W, Song X, Gao R, Wang J. Phosphodiesterase 4D promotes angiotensin II-induced hypertension in mice via smooth muscle cell contraction. Commun Biol. 2022 Jan 20;5(1):81. doi: 10.1038/s42003-022-03029-0. PMID: 35058564; PMCID: PMC8776755.

Anti-STON1-GTF2A1L: Manufacturer-verified for use in: WB, IHC. Immunofluorescence staining in human RPE cells demonstrated specific colocalization with the same markers as those identified using the in-house developed and knock-out validated Stonin1 antibody in C2C12 cells. Used only in supplemental Fig. 2G.

Anti-STON2: Manufacturer-verified for use in: IF and WB. Relevant citation: Diril MK, Wienisch M, Jung N, Klingauf J, Haucke V. Stonin 2 is an AP-2-dependent endocytic sorting adaptor for synaptotagmin internalization and recycling. Dev Cell. 2006 Feb;10(2):233-44. doi: 10.1016/j.devcel.2005.12.011. PMID: 16459302.

Anti-mouse Stonin1: The in-house developed rabbit polyclonal antibody underwent validation through Western blot and immunofluorescence analyses using Ston1 knockout mouse fibroblasts. Relevant citation: Feutlinske F, Browarski M, Ku MC, Trnka P, Waiczies S, Niendorf T, Stallcup WB, Glass R, Krause E, Maritzen T. Stonin1 mediates endocytosis of the proteoglycan NG2 and regulates focal adhesion dynamics and cell motility. Nat Commun. 2015 Oct 5;6:8535. doi: 10.1038/ncomms9535. PMID: 26437238; PMCID: PMC4600748.

Anti-Vinculin: Manufacturer-verified for use in: WB, IF. Relevant citation: Lock JG, Jones MC, Askari JA, Gong X, Oddone A, Olofsson H, Göransson S, Lakadamyali M, Humphries MJ, Strömblad S. Reticular adhesions are a distinct class of cell-matrix adhesions that mediate attachment during mitosis. Nat Cell Biol. 2018 Nov;20(11):1290-1302. doi: 10.1038/s41556-018-0220-2. Epub 2018 Oct 22. PMID: 30361699.

Anti-αAdaptin: Manufacturer-verfied for use in: Flow cytometry, ICC/IF. Relevant citation: Moulay G, Lainé J, Lemaître M, Nakamori M, Nishino I, Caillol G, Mamchaoui K, Julien L, Dingli F, Loew D, Bitoun M, Leterrier C, Furling D, Vassilopoulos S. Alternative splicing of clathrin heavy chain contributes to the switch from coated pits to plaques. J Cell Biol. 2020 Sep 7;219(9):e201912061. doi: 10.1083/jcb.201912061. PMID: 32642759; PMCID: PMC7480091.

Anti-mouse CD51 (αV Integrin): Manufacturer-verified for use in: Flow cytometry. The antibody was validated with immunofluorescence following siRNA-mediated knockdown (ON-TARGETplus Mouse Itgav (16410) siRNA - SMARTpool) of the target protein in C2C12 cells.

Anti-αV Integrin: Manufacturer-verified for use in: WB, IP, IF, ELISA. Relevant citation: Antoniades I, Kyriakou M, Charalambous A, Kalalidou K, Christodoulou A, Christoforou M, Skourides PA. FAK displacement from focal adhesions: a promising strategy to target processes implicated in cancer progression and metastasis. Cell Commun Signal. 2021 Jan 7;19(1):3. doi: 10.1186/s12964-020-00671-1. PMID: 33413438; PMCID: PMC7791867.

Anti-β-actin: Manufacturer-verified for use in: WB, IP, IF, IHC. Relevant citation: Wallroth A, Koch PA, Marat AL, Krause E, Haucke V. Protein kinase N controls a lysosomal lipid switch to facilitate nutrient signalling via mTORC1. Nat Cell Biol. 2019 Sep;21(9):1093-1101. doi: 10.1038/s41556-019-0377-3. Epub 2019 Aug 26. PMID: 31451768.

Anti-β5 Integrin: Manufacturer-verified for use in: WB, IP, IF. Relevant citation: Leyton-Puig D, Isogai T, Argenzio E, van den Broek B, Klarenbeek J, Janssen H, Jalink K, Innocenti M. Flat clathrin lattices are dynamic actin-controlled hubs for clathrin-mediated endocytosis and signalling of specific receptors. Nat Commun. 2017 Jul 13;8:16068. doi: 10.1038/ncomms16068. PMID: 28703125; PMCID: PMC5511353.

Abbreviations: WB, western blotting; IP, immunoprecipitation; IF, immunofluorescence; ICC, immunocytochemistry; IHC, immunohistochemistry

Eukaryotic cell lines

Policy information about cell lines and Sex and Gender in Research

Cell line source(s)	C2C12 (#CRL-1772), hTERT RPE1 (#CRL-4000) and HEK293T (# CRL-3216) cells were originally obtained from ATCC.
Authentication	Cell lines from ATCC are regularly authenticated by STR profiling.
Mycoplasma contamination	Cell lines were regularly tested for mycoplasma contamination and were not contaminated.
Commonly misidentified lines (See ICLAC register)	There were no commonly misidentified cell lines used in this study.

Plants

Seed stocks	*Report on the source of all seed stocks or other plant material used. If applicable, state the seed stock centre and catalogue number. If plant specimens were collected from the field, describe the collection location, date and sampling procedures.*
Novel plant genotypes	*Describe the methods by which all novel plant genotypes were produced. This includes those generated by transgenic approaches, gene editing, chemical/radiation-based mutagenesis and hybridization. For transgenic lines, describe the transformation method, the number of independent lines analyzed and the generation upon which experiments were performed. For gene-edited lines, describe the editor used, the endogenous sequence targeted for editing, the targeting guide RNA sequence (if applicable) and how the editor was applied.*
Authentication	*Describe any authentication procedures for each seed stock used or novel genotype generated. Describe any experiments used to assess the effect of a mutation and, where applicable, how potential secondary effects (e.g. second site T-DNA insertions, mosiacism, off-target gene editing) were examined.*
